# Supplementary material for: Laser-induced graphitization of polydopamine leads to enhanced mechanical performance while preserving multifunctionality
Source: Nat Commun. 2020 Sep 24;11:4848. doi: 10.1038/s41467-020-18654-8 (PMC7515926; doi:10.1038/s41467-020-18654-8)
Supplement: Supplementary file 1 — Supplementary Information [file 41467_2020_18654_MOESM1_ESM.pdf]

Supplementary Information

Laser-induced graphitization of polydopamine leads to enhanced mechanical performance while preserving multifunctionality

Kyueui Lee+, Minok Park+, Katerina G. Malollari, Jisoo Shin, Sally M. Winkler, Yuting Zheng, Jung Hwan Park, Costas P. Grigoropoulos*, and Phillip B. Messersmith*

+These authors contributed equally.

Supplementary Figures and Tables

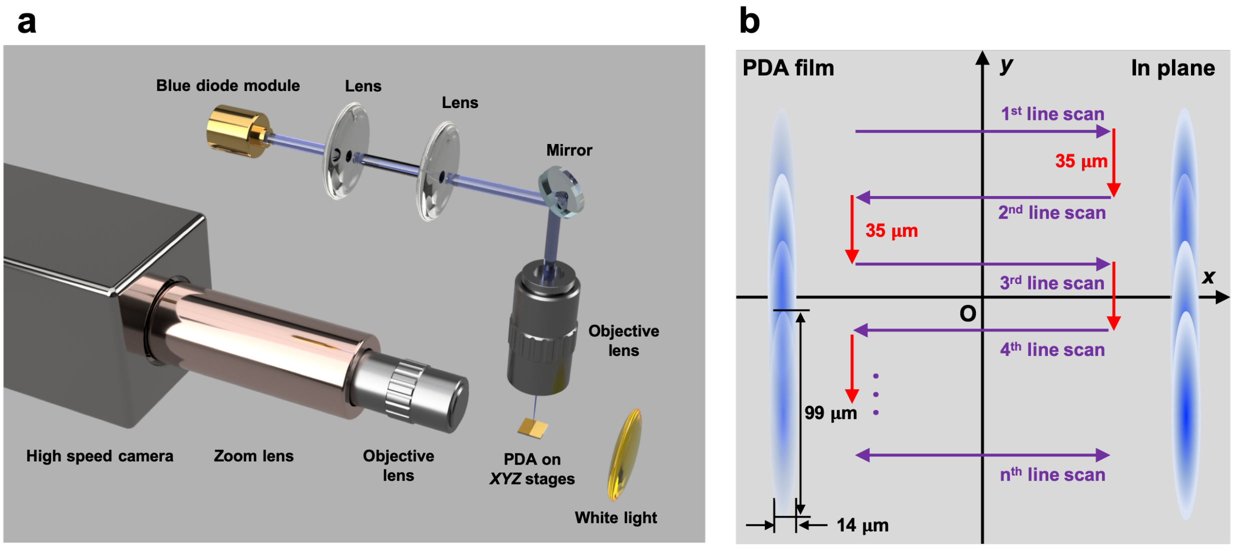

Supplementary Figure 1. The schematics of (a) the optical setup for the BLA process, and (b) the raster scanning method to anneal the PDA film.

We utilized the fundamental (elliptical) laser source to demonstrate the capability of large area processing via a line beam shape. Moreover, an elliptically shaped beam profile with 99 μm long axis and 14 μm short axis is employed to obtain sufficient laser intensities (kW cm^-2) within the output of a single diode laser in order to ensure the temperature rise.

Laser Power (W)	Laser Intensity (kW cm^-2)
0.9	20.7
1.2	27.6
1.9	43.6
2.4	55.1
2.9	66.6

Supplementary Table 1. Laser conditions in intensity (kW cm^-2)

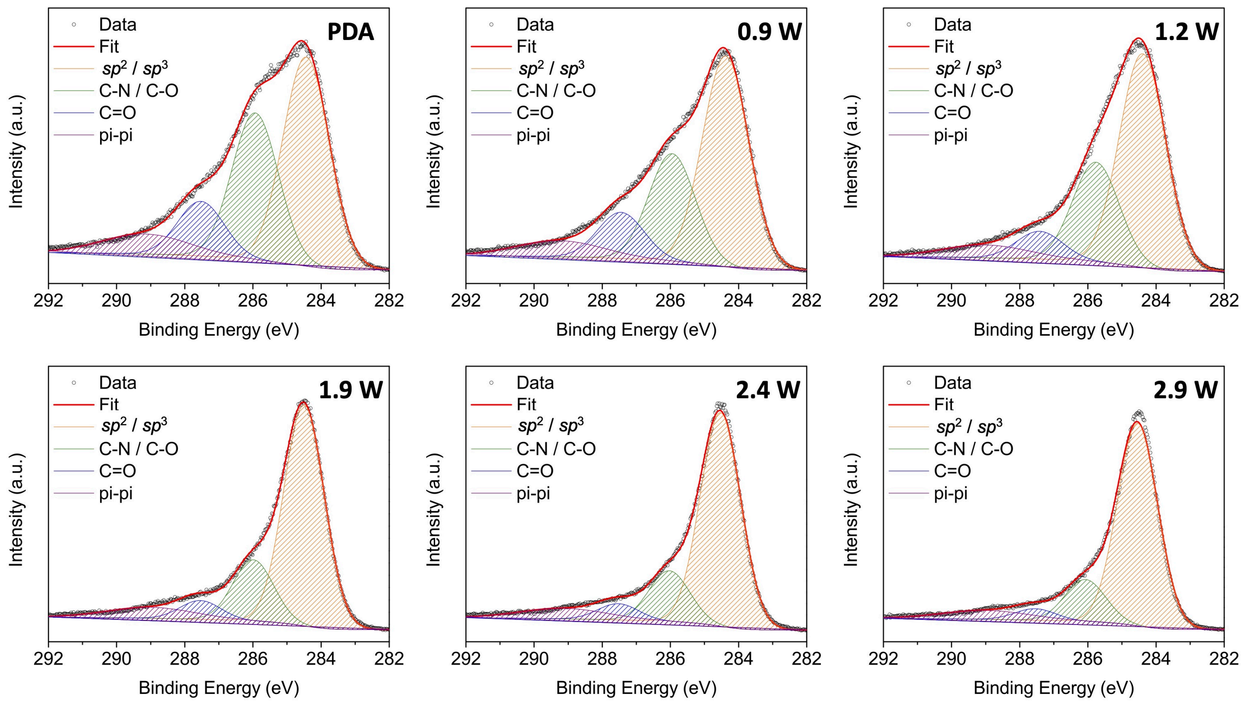

Supplementary Figure 2. Deconvoluted XPS high-resolution *C1s* spectra taken from the PDA and the LAPDA films obtained at different laser powers of 0.9 W, 1.2 W, 1.9W, 2.4 W, and 2.9 W. Source data are provided as a Source Data file.

power (W)	composition (at.%)			
	sp^2 / sp^3	C-N / C-O	C=O	π-π
-	47	33	12	8
0.9	53	27	12	8
1.2	59	27	8	7
1.9	68	19	6	7
2.4	71	17	5	7
2.9	74	15	4	7

Supplementary Table 2. The atomic percentages of functional groups composing the pristine PDA and the LAPDA films. Source data are provided as a Source Data file.

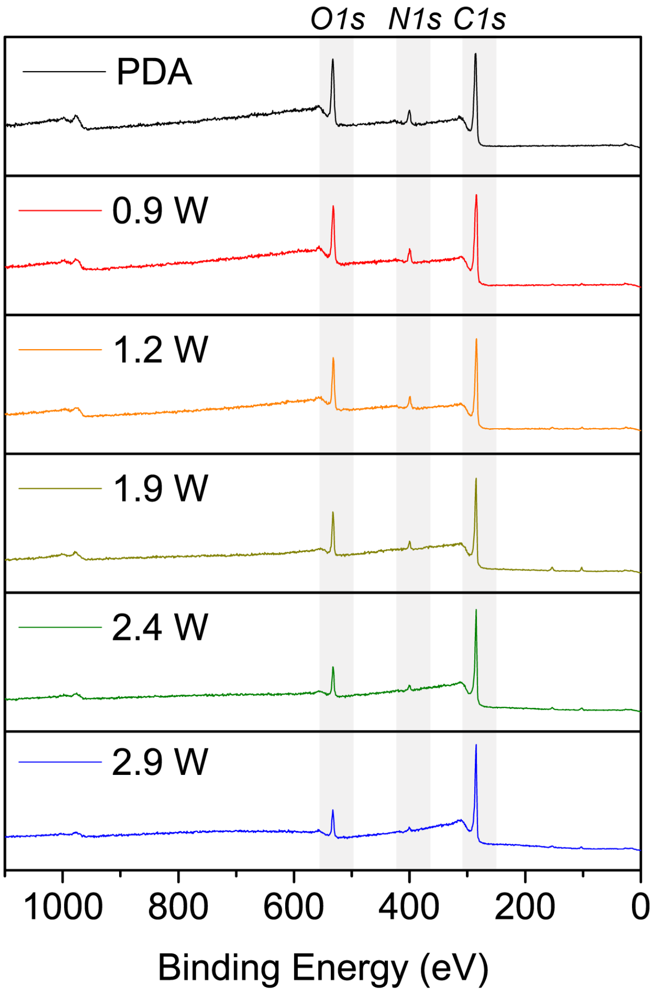

Supplementary Figure 3. The XPS survey spectra collected from the pristine PDA and the LAPDA films. Source data are provided as a Source Data file.

power (W)	composition (at.%)		
	C1s	***N1s***	***O1s***
-	72	7	22
0.9	75	7	18
1.2	75	7	18
1.9	79	5	17
2.4	84	4	12
2.9	87	3	11

Supplementary Table 3. The atomic percentages of elements composing the pristine PDA and the LAPDA films. Source data are provided as a Source Data file.

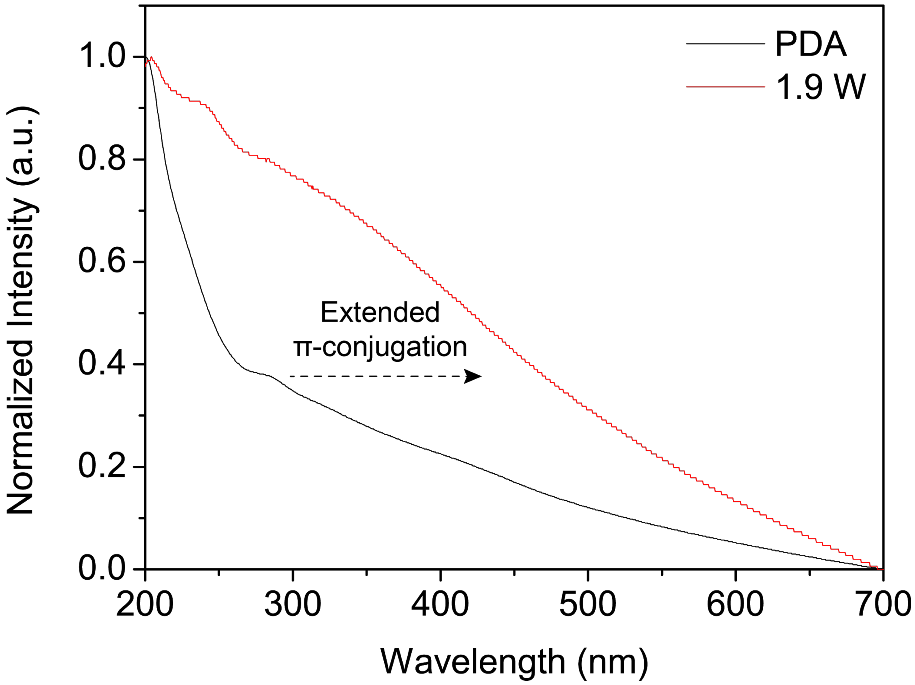

Supplementary Figure 4. The normalized UV-VIS spectra of the pristine PDA and the LAPDA at 1.9 W. Source data are provided as a Source Data file.

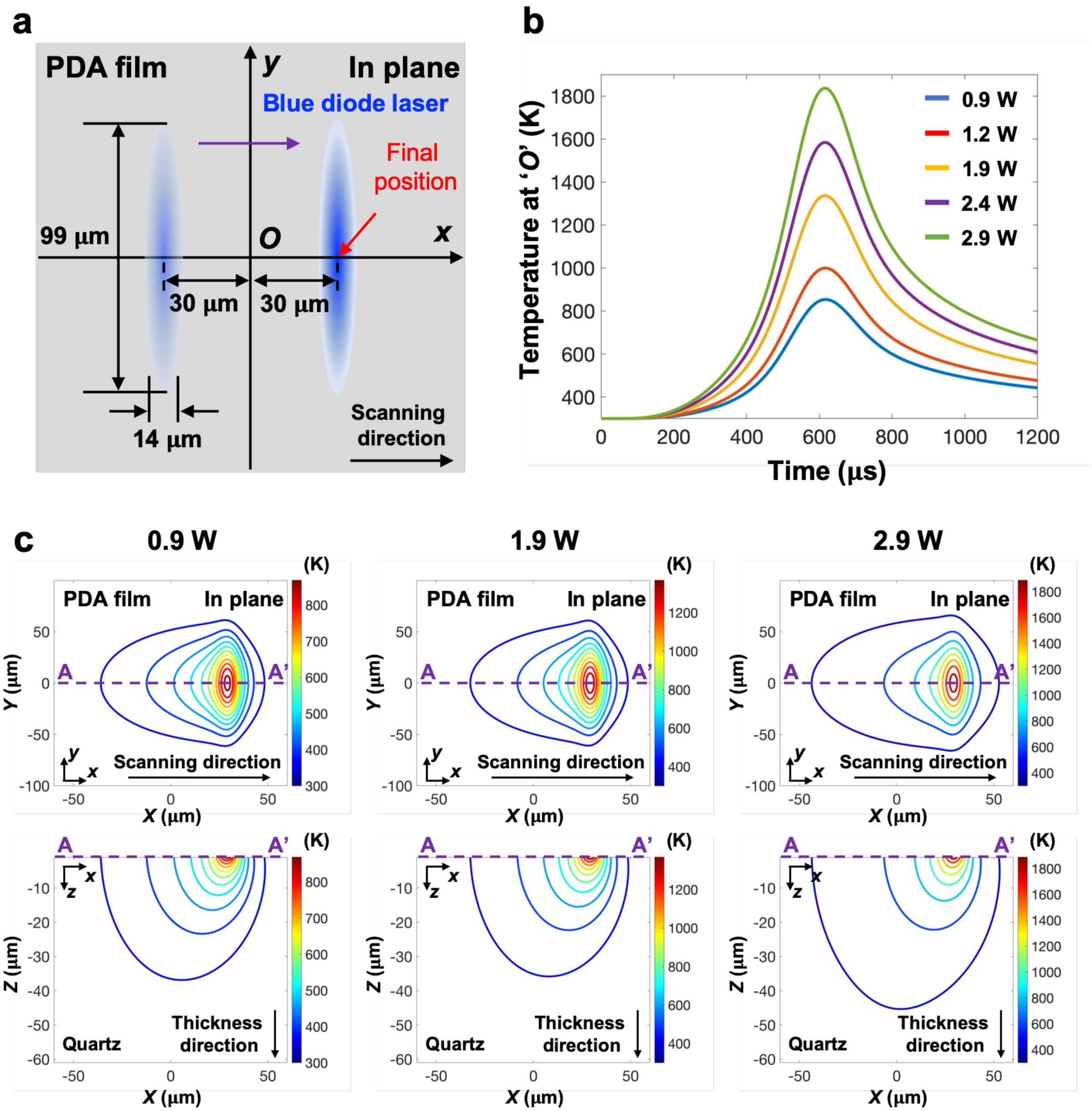

Supplementary Figure 5. (a) Schematic of numerical simulations, (b) time-dependent temperature profiles at *O* under different laser powers, and (c) contours of transient temperature fields at 0.9 W, 1.9 W, (c) 2.9 W. Note that +*x* direction is the scanning direction. Source data are provided as a Source Data file.

When a continuous wave laser is irradiated on absorbing media, the absorbed energy contributes to increasing the temperatures, which can be experimentally probed and modeled by a heat conduction equation[1,2,3]. Hence, to estimate the transient temperature field of the flat PDA film, a

three-dimensional heat conduction equation was numerically solved by the finite difference method[4]. Because the flat PDA film is semi-transparent at 440 nm wavelength and its thickness (approximately 100 nm) is much thinner compared to the thermal diffusion length (approximately 20 μm at dwelling time of 280 μs and scanning speed of 50 mm s^-1), the entire PDA film is volumetrically heated by the blue diode laser. Furthermore, most of the heat is dissipated into the substrate as the thermal penetration depth is much bigger than the film thickness. Therefore, the pristine PDA film was modeled as a simplified two-dimensional layer whereas the quartz substrate was modeled in three-dimensional[4]. Furthermore, all surfaces were modeled as an adiabatic boundary condition, as presented below.

$$\rho_{PDA} C_{p,PDA} \frac{dT_{PDA}}{dt} = k_{PDA} \nabla^2 T_{PDA} + \frac{1}{\delta} \int_0^{\delta} \alpha_{PDA} I(x, y, z, t)\, dz \qquad (1)$$

$$\rho_{SiO2} C_{p,SiO2} \frac{dT_{SiO2}}{dt} = k_{SiO2} \nabla^2 T_{SiO2} \qquad (2)$$

$$k \frac{\partial T}{\partial r}\bigg|_{all\ surfaces} = 0 \qquad (3)$$

where, ρ is the density, C_p is the heat capacity, and T is the temperature at time t, δ is the thickness of the PDA film, α is absorbance of the PDA, and I is the laser intensity.

The numerical simulation performed accounts only for the laser absorption and heat diffusion to characterize the upper bound of the transient temperature of the PDA film under different laser powers (0.9 W, 1.2 W, 1.9 W, 2.4 W, and 2.9 W). The corresponding absorbed laser powers on PDA films are 0.05 W, 0.06 W, 0.1 W, 0.12 W, and 0.15 W, respectively. The modeling of the phase change mechanism from amorphous to poly-crystalline graphitization was not included herein. The actual laser profile measured by the knife-edge experiment was utilized for

the simulation. As the structure of the PDA is expected to be comparable with that of melanin, thermal and physical properties of melanin (thermal conductivity ~ 0.63 W m^-1 K^-1, density ~ 2000 kg m^-3 and heat capacity ~2500 J kg^-1 K^-1) were alternatively utilized[5], while properties of quartz were found in literature[6].

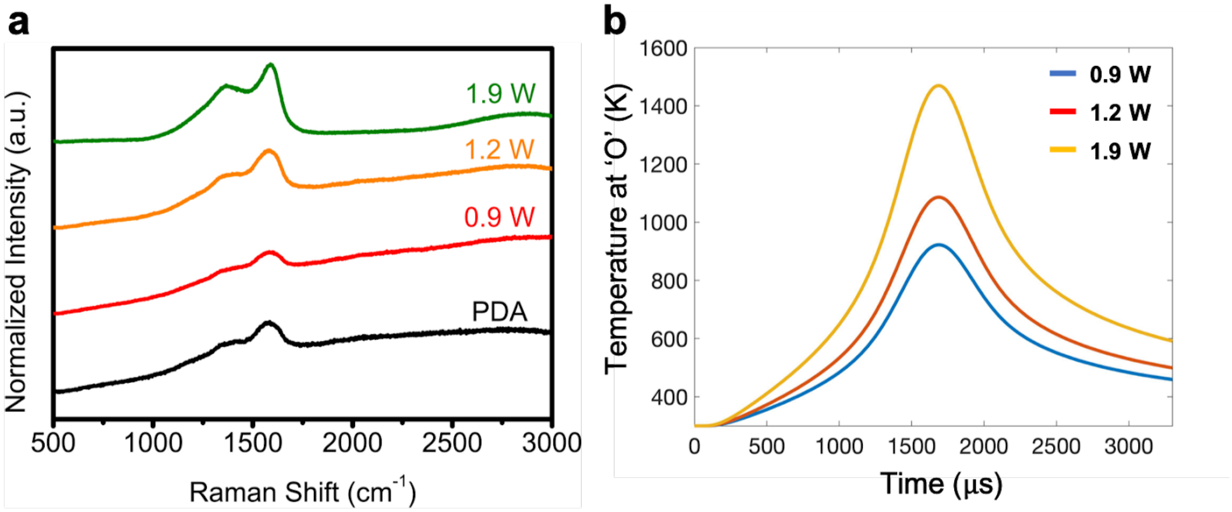

Supplementary Figure 6. (a) Graphitization level of each LAPDA film with 18 mm s^-1 scanning speed. (b) Time-dependent temperature profiles at *O* under different laser powers. Schematic of numerical simulations is shown in Supplementary Figure 5a. Source data are provided as a Source Data file.

There are two possible mechanisms in the UV, and to a lesser extent in the near UV ranges that may affect graphitization (i.e., cross-linking between building blocks). The relative contributions of these mechanisms depend upon the imparted wavelength, pulse duration (or dwell time), and the material properties. Hence, the LAPDA process was scrutinized to verify which process leads to graphitization at the blue wavelength (440 nm) by varying the scanning speed. If the dominant mechanism was photochemical, similar results would be obtained by reducing the laser power and increasing the exposure time (dwell time), as typical in UV curing of polymers. Furthermore, multi-photon effects are unlikely for the relatively low photon fluxes in CW laser processing.

Specifically, experiments under the same laser intensities but with lower scanning speed (18 mm s^-1 equivalent to 778 μs dwell time) were shown in Supplementary Figure 6. Based on Raman spectroscopy in Supplementary Figure 6a, ~2.8 times more dwell time with 0.9 W did not have a substantial effect on the graphitization, but laser powers higher than 1.2 W started inducing partial

graphitization. To further put the estimated temperature in perspective, numerical simulations at 0.9 W, 1.2 W, and 1.9 W with 18 mm s^-1 were performed and presented in Supplementary Figure 6b. As a result, the maximum temperature under 0.9 W was estimated at 922 K (below 1000 K), and above 1000 K at 1.2 W, and 1.9 W, which is consonant with the predictions and experimental results of the 50 mm s^-1 annealing. Therefore, when combining all experimental and numerical analysis, it is believed that the main mechanism of the LAPDA process rendered by BLA and leading to partial graphitization is photothermal rather than photochemical.

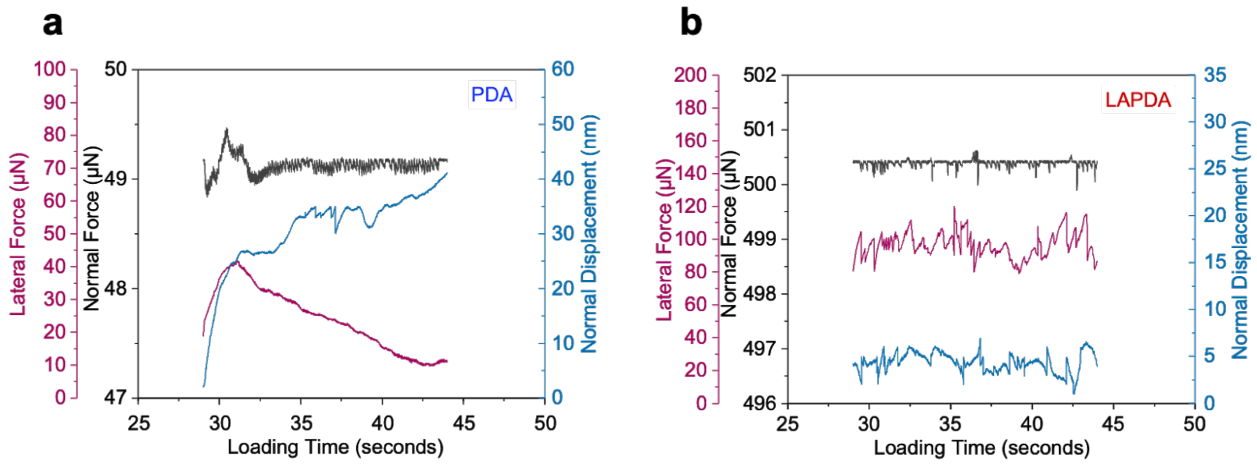

Supplementary Figure 7. Representative raw data illustrating the lateral load, normal load and normal displacement as a function of loading time for (a) pristine PDA at 50 µN and (b) LAPDA at 500 µN. Source data are provided as a Source Data file.

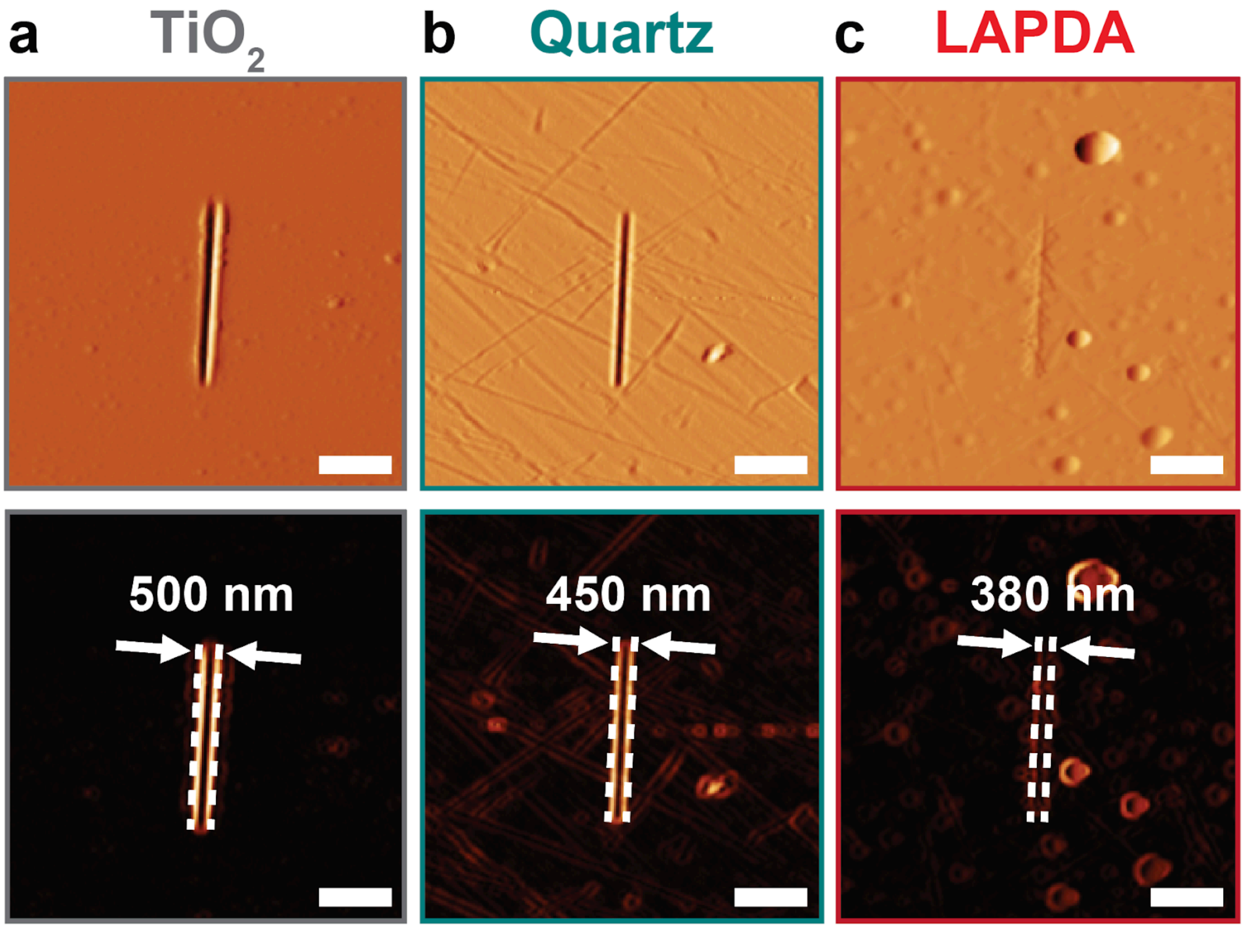

Supplementary Figure 8. (a) Scratch tracks for TiO2, quartz and the LAPDA after the scratch measurements. (b) Analyzed with Gwyddion software for (a). The scale bar is 2 µm.

The hardness test utilizes the residual scratch width after the indenter is removed reflecting the permanent deformation resulting from scratching and not the instantaneous state of combined elastic and plastic deformation of the surface. The scratch hardness number can be calculated by dividing the applied normal load by the projected area of scratching contact by using the following equation[7]:

$$H=8P\ \pi^{-1}\ w^{-2}$$

where P = normal force, and w = scratch width.

Representative images of the scratch tracks after the test was performed are shown in Figure 2. TiO_2 exhibits the highest width (500 nm in this case) followed by quartz and finally LAPDA with 450 nm and 380 nm respectively, resulting in calculated hardness values of $H_{TiO2} = 4.6 \pm 0.4$ GPa, $H_{Quartz} = 6.2 \pm 0.9$ GPa, and $H_{LAPDA} = 6.6 \pm 1.0$ GPa. All the hardness values are comparable, with LAPDA exhibiting the highest, which is also evident in the profile images. The above values were taken as average values out of four measured widths for each sample.

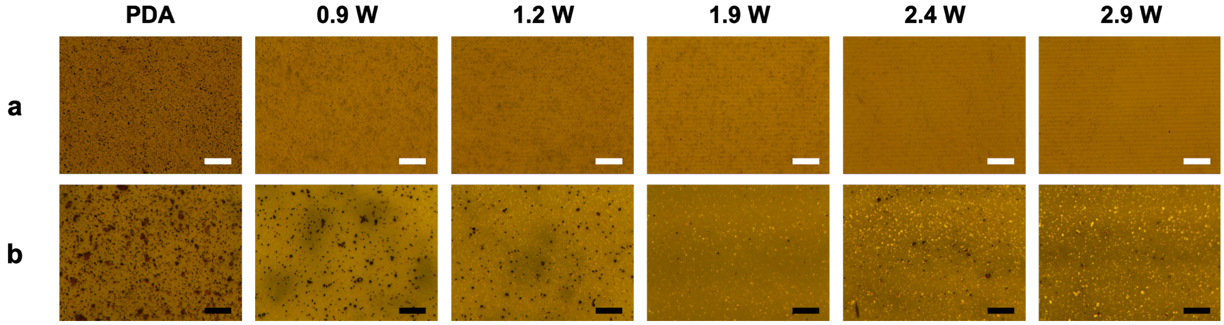

Supplementary Figure 9. Optical microscope images of the pristine PDA and the LAPDA films at 0.9 W, 1.2 W, 1.9 W, 2.4 W, and 2.9 W. Images in (a) are 10× optical images (white scale bar 100 µm), and those in (b) are 100× optical images (black scale bar 10 µm).

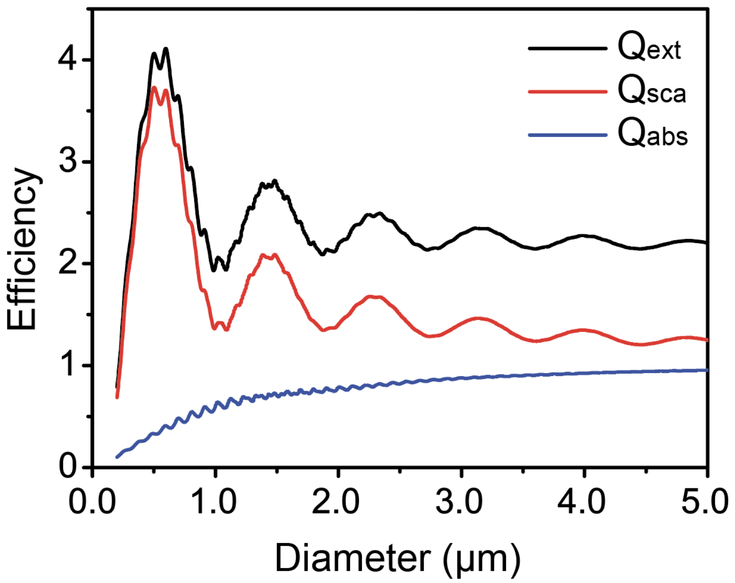

Supplementary Figure 10. Calculated extinction (Q_{ext}), absorption (Q_{abs}), and scattering efficiency (Q_{sca}) of the PDA NP by Mie theory modeling.

We calculated the scattering and absorption efficiency of the PDA particles based on Mie theory[8] using the complex refractive index of PDA (n = 1.52 and k = 0.02 at 440 nm)[9], assuming that agglomerated NPs could be treated as a single spherically shaped particle. PDA particle of 0.25 μm diameter has 0.16 of absorption efficiency that increases to 0.95 for 5 μm diameter particle. On the other hand, the absorptance of the flat PDA film having 100 nm thickness (Figure 3a) has only 0.05.

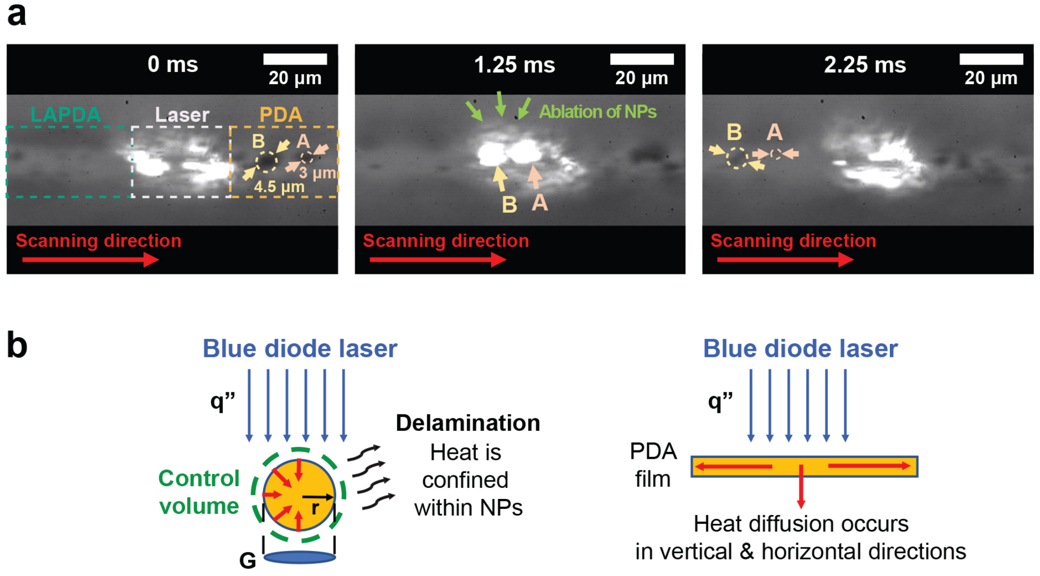

Supplementary Figure 11. Light-induced PDA NPs removal process. (a) Selected snapshots captured from the high-speed video with 8000 fps, and (b) schematic of the ablation mechanism of PDA NPs (left), and that of the heat diffusion in case of the PDA film (right).

In order to verify the ablation mechanism for PDA NPs, the high-speed recording system with 8000 fps (125 µs acquisition per frame) was utilized to probe *in-situ* the removal process of NPs under 1.9 W laser power and 50 mm s^-1 scanning speed conditions (Supplementary Figure 11a). Specifically, the NP marked as A has a size of 3 µm, and the NP indicated as B has a size of 4.5 µm. During the BLA, these aggregated NPs were being ablated as shown in Supplementary Figure 11a. After the processing, NPs in group A were totally delaminated, but a trace of NPs in group B still remained. Through these observations, a lumped capacitance analysis was used to estimate the order of magnitude of the annealing temperature for the PDA NPs during the BLA process.

From the conservation of the energy at the time instant t, the energy balance equation on a control volume encompassing the NP, as shown in Supplementary Figure 11b (left), can be expressed as below.

$$Q_{abs} G q_s^{"} - [h(T - T_\infty) + \varepsilon\sigma(T^4 - T_\infty^4)]A_s = \rho V C_p \frac{dT}{dt} \tag{4}$$

where, Q_{abs} is the absorption efficiency, G is the cross-section of the NP, q_s is the laser fluence, ρ is the density, σ is the Stefan-Boltzmann constant, A_s and V are the surface area and the volume of the NP, C_p is the heat capacity, and T is the transient temperature at time t. As in Supplementary Figure 5, we adopt thermal conductivity (~ 0.63 W m^-1 K^-1), density (~ 2000 kg m^-3) and heat capacity (~ 2500 J kg^-1 K^-1) of melanin[2] for PDA.

The Biot number, Bi = h Lc k^-1, where h is the heat transfer coefficient, Lc is the characteristic length, taken as the diameter of the particle, and k the thermal conductivity. While h cannot be measured directly, this dimensionless number is very small, ~ 0.001 even when a very high value of h of 1000 W m^-2 K^-1 is assumed as upper bound. Consequently, the spatial distribution of the transient temperature field within the particle can be neglected. Hence, it is legitimate to use the lumped capacitance analysis for this problem and neglecting radiative and convective losses, the above equation can be reduced as below:

$$Q_{abs} G q_s^{"} \approx \rho V C_p \frac{dT}{dt} \tag{5}$$

From the above equation, the absorbed heat is mainly utilized to increase the temperature of the NP. With annealing duration of 280 μs, the estimated temperature of the PDA particles derived from the equation is ~ 2900 K for A: ~ 3 μm size, and ~ 2100 K for B: ~ 4.5 μm size. This indicates that the incident laser results in higher temperature in the particles due to high absorption efficiency. In contrast, deposited energy in the flat PDA film diffuses into the substrate and ~ 1300 K peak temperature is driven by laser power of 1.9 W according to the numerical results in

Supplementary Figure 5. Therefore, particles could be selectively ablated from the surface, without damaging the film.

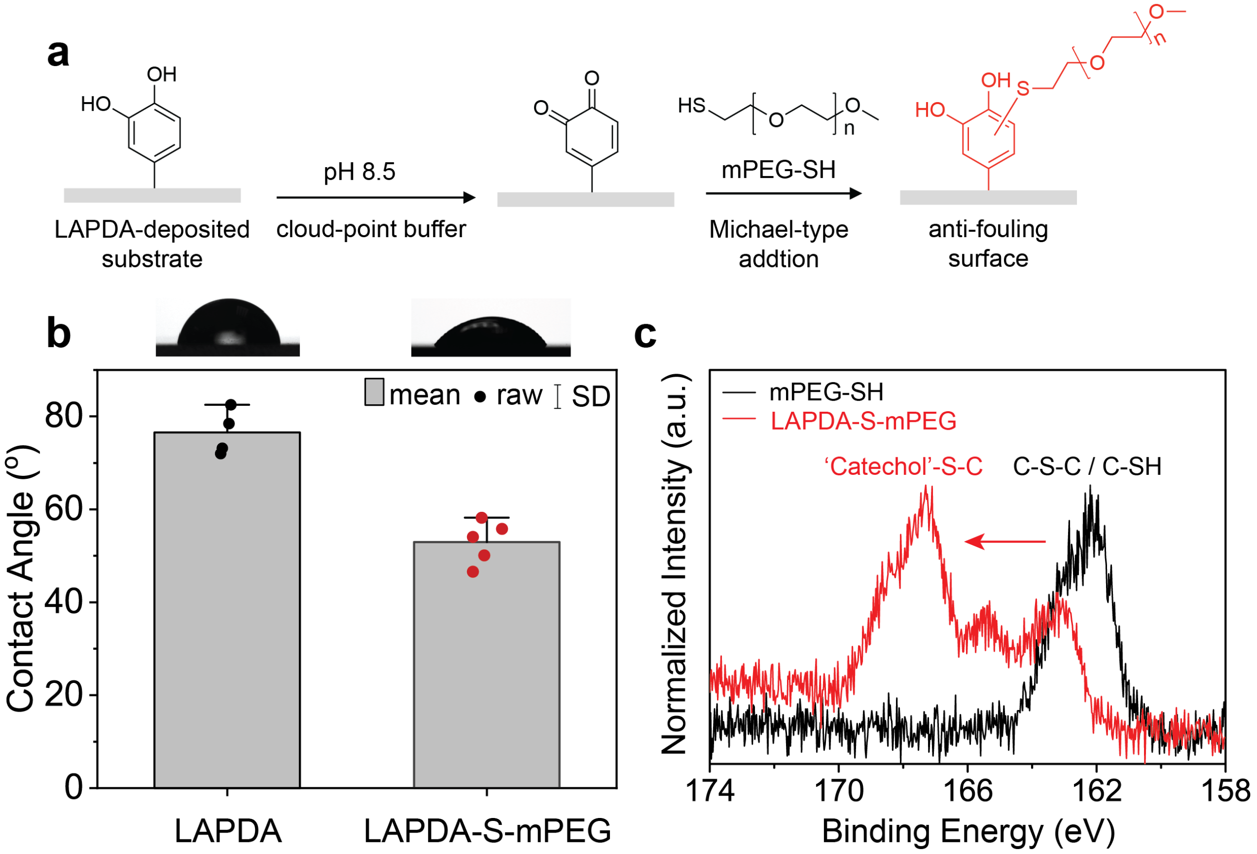

Supplementary Figure 12. Grafting of mPEG-SH on the LAPDA coated substrate. (a) Schematic of chemical mechanisms of the post-modification, (b) the contact angles of LAPDA and PEGylated LAPDA surfaces. Bars represent mean values and original data points overlaid as black or red dots (n = 5, SD indicates standard deviation); error bars represent standard deviation. (c) the high-resolution *S2p* spectra of the pristine mPEG-SH (black) and PEGylated LAPDA. Source data are provided as a Source Data file.

To achieve the antifouling surfaces, a representative anti-fouling material, methoxy polyethylene glycol thiol (mPEG-SH), was grafted onto the PDA and LAPDA surfaces as previous reported[10]. Briefly, the PDA (or the LAPDA) coated substrate (quartz) was immersed into the solution of 1 mM mPEG-SH (MW 5000, Laysan Bio Inc, USA) in 0.6 KCl and 10 mM bicine (pH 8.5 – pH was adjusted by adding 1 M NaOH), and incubated in the oven for 6 hours at 55 °C (Supplementary Figure 12a). Subsequently, the reaction solution was replaced with deionized water 5 times to wash

away unreacted mPEG-SH. For contact angle and XPS measurements, the substrate was gently side-blotted with filter paper (Whatman, UK) to remove residual water on the surface, and dried with nitrogen gas before use. For bacterial studies, the substrate was transferred to the phosphate-buffered saline (1X PBS, pH 7.4) and stored at room temperature until use.

The mPEG-SH conjugation on the LAPDA surface was confirmed by the three pieces of evidence. (1) The contact angle of the PEGylated LAPDA was significantly reduced compared to the unmodified LAPDA due to the hydrophilic PEG group (Supplementary Figure 12b). (2) A sulfur signal from the grafted thiol groups was obviously detected from the high-resolution *S2p* XPS spectrum of PEGylated LAPDA (Supplementary Figure 12c). (3) The binding energy of the *S2p* XPS spectrum of PEGylated LAPDA was positively shifted compared to that of mPEG-SH, due to the covalent conjugation between thiols and catechols (Supplementary Figure 12c).

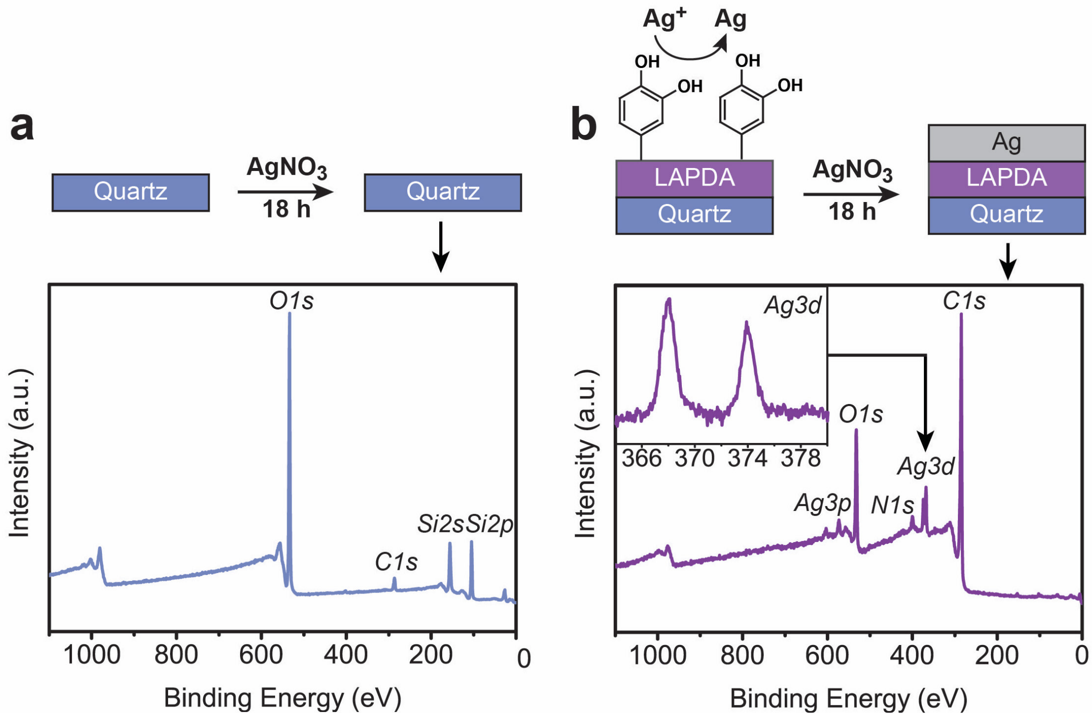

Supplementary Figure 13. Demonstration of catechol functionality through electroless metallization on LAPDA. The schematic of 50 mM silver nitrate solution treatment on (a) bare quartz and (b) LAPDA on quartz; and their corresponding X-ray photoelectron spectroscopy analyses were described on the bottom of each scheme. The high-resolution *Ag3d* data was additionally collected from the treated LAPDA substrate, and presented at the left top of the corresponding plot. Source data are provided as a Source Data file.

Inherent functionality in LAPDA was further confirmed by electroless metallization, which is another well-defined ability of catechols where metal ions are reduced, resulting in equivalent metallic layers[11]. For this, LAPDA at 1.9 W on quartz was dipped into the aqueous silver nitrate solution (50 mM) for 18 hours under mild shaking condition. Subsequently, the substrate was washed with deionized water, and dried with nitrogen gas. A bare quartz which underwent the same processes was also prepared for the comparison. To confirm the metallic layer (i.e. silver), we performed XPS analysis on both substrates. Whereas no relevant peaks were observed on the bare quartz (Supplementary Figure 13a), there were strong silver peaks on the LAPDA surface (Supplementary Figure 13b). This indicates that the silver ions were selectively reduced on the

LAPDA surface, demonstrating that the inherent catechol functionality is preserved on the LAPDA surface.

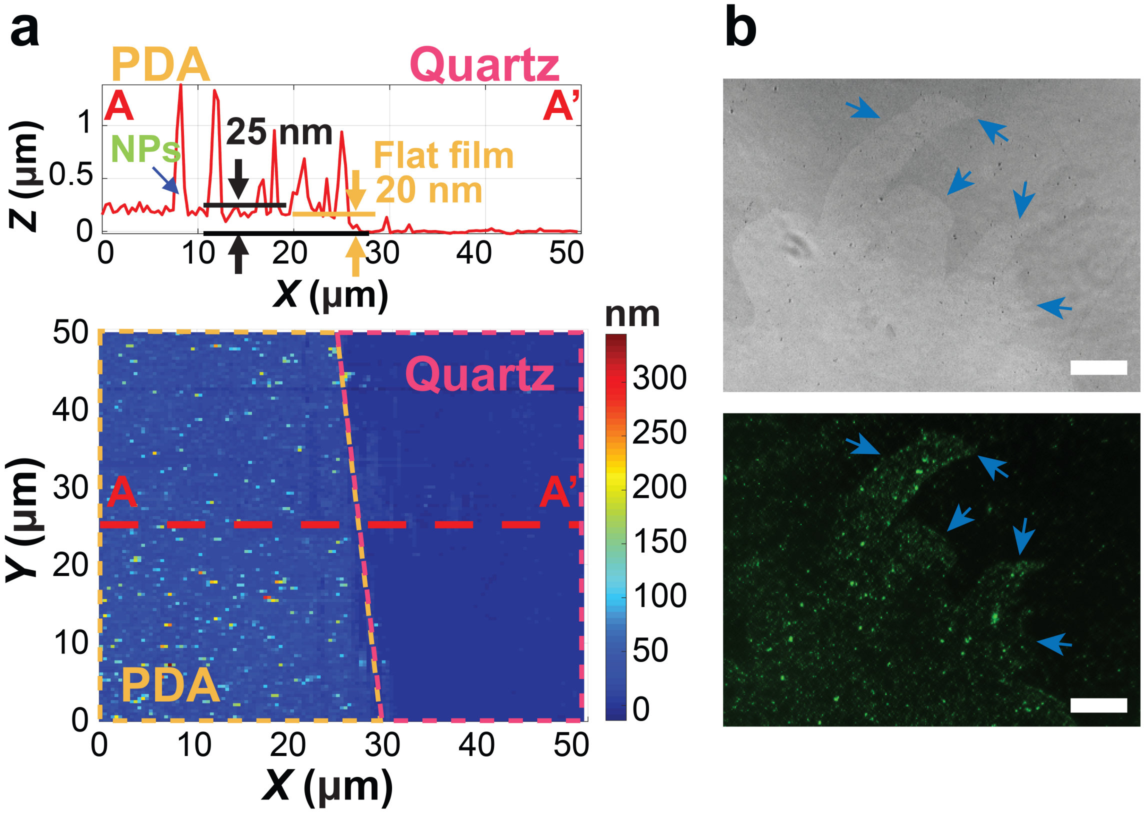

Supplementary Figure 14. (a) AFM image with line-scan profile of pristine PDA deposited for 2 hours, showing 25 nm average thickness (20 nm flat PDA film with 20 nm roughness). (b) The bright-field (upper) and fluorescence (lower) images of bacterial attachment on the PEGylated PDA film after 24 hours of incubation. The blue arrows indicate bacterial accumulation on sites of mechanical damage in the PEGylated PDA coating. The scale bar is 200 μm. Source data are provided as a Source Data file.

In addition to 248 nm average thickness of PDA film (Figure 3a), a thinner PDA layer that is as smooth as the LAPDA was further prepared by reducing the coating time (Supplementary Figure 14a). The PDA-coated substrate was subsequently PEGylated by following the protocols described in the Methods section. It was found that the physical delamination occurred in the thin film (Supplementary Figure 14b) as comparable as in the thick film (Figure 4b), which similarly

resulted in the partial failure of antifouling from the bacterial adhesion. Given that the PEGylated LAPDA film sharing similar thickness and smoothness of the thin PDA film showed even better antifouling performance (Figure 4a) compared to the thick PEGylated PDA film (Figure 4b), the above result once again highlights the positive effect of mechanically strengthened LAPDA film.

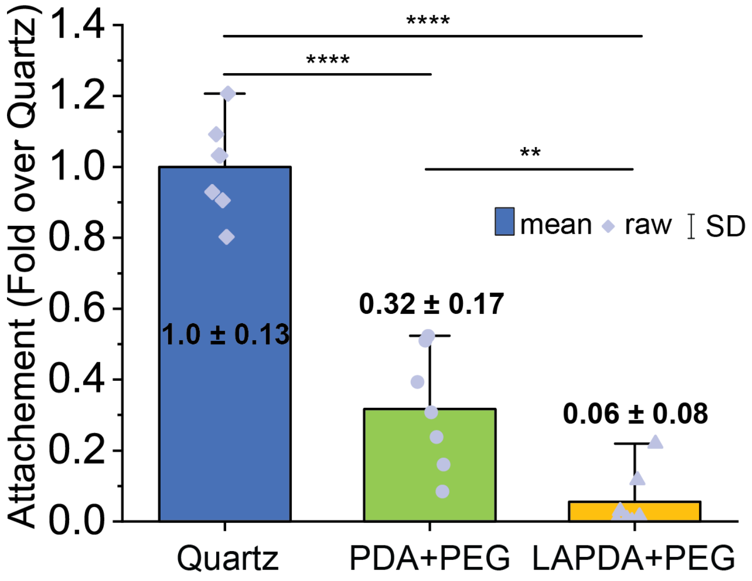

Supplementary Figure 15. Quantified bacterial attachment after 24 hours of incubation in three different surfaces: bare quartz, PEGylated PDA, and PEGylated LAPDA. All the values were normalized to the average area of bacterial attachment in bare quartz. Bars represent mean values and original data points overlaid as purple dots; error bars represent standard deviation. P values less than 0.01 and 0.0001 shown as two and four asterisks, respectively (n = 7). Source data are provided as a Source Data file.

Supplementary References

1. Grigoropoulos CP, Dutcher WE, Jr., Emery AF. Experimental and Computational Analysis of Laser Melting of Thin Silicon Films. *Journal of Heat Transfer* **113**, 21-29 (1991).
2. Xu X, Taylor SL, Park HK, Grigoropoulos CP. Transient heating and melting transformations in argon-ion laser irradiation of polysilicon films. *Journal of Applied Physics* **73**, 8088-8096 (1993).
3. Grigoropoulos CP, Rostami AA, Xu X, Taylor SL, Park HK. Transient reflectivity measurements and heat transfer modeling in laser annealing of semiconductor films. *International Journal of Heat and Mass Transfer* **36**, 1219-1229 (1993).
4. Webber HC, Cullis AG, Chew NG. Computer simulation of high speed melting of amorphous silicon. *Applied Physics Letters* **43**, 669-671 (1983).
5. Crippa PR, Viappiani C. Photoacoustic studies of non-radiative relaxation of excited states in melanin. *European Biophysics Journal* **17**, 299-305 (1990).
6. Grigoropoulos CP, Buckholz RH, Domoto GA. A Thermal Instability in the Laser-Driven Melting and Recrystallization of Thin Silicon Films on Glass Substrates. *Journal of Heat Transfer* **109**, 841-847 (1987).
7. *ASTM International.* G171-03(2017) Standard Test Method for Scratch Hardness of Materials Using a Diamond Stylus. West Conshohocken, PA, 2017.
8. Bohren CF, Huffman DR, *Absorption and Scattering of Light by Small Particles*, Wiley, New York (1983).
9. Vega M, Martín del Valle EM, Pérez M, Pecharromán C, Marcelo G. Color Engineering of Silicon Nitride Surfaces to Characterize the Polydopamine Refractive Index. *ChemPhysChem* **19**, 3418-3424 (2018).

10. Sileika TS, Kim H-D, Maniak P, Messersmith PB. Antibacterial Performance of Polydopamine-Modified Polymer Surfaces Containing Passive and Active Components. *ACS Applied Materials & Interfaces* **3**, 4602-4610 (2011).

11. Lee H, Dellatore SM, Miller WM, Messersmith PB. Mussel-Inspired Surface Chemistry for Multifunctional Coatings. *Science* **318**, 426 (2007).
