# Supplementary material for: Laser-induced graphitization of polydopamine leads to enhanced mechanical performance while preserving multifunctionality
Source: Nat Commun. 2020 Sep 24;11:4848. doi: 10.1038/s41467-020-18654-8 (PMC7515926; doi:10.1038/s41467-020-18654-8)
Supplement: Supplementary file 3 — Description of Additional Supplementary Files [file 41467_2020_18654_MOESM3_ESM.pdf]

Description of Additional Supplementary Files

File Name: Supplementary Movie 1
Description: The post-processing of the PDA film via BLA process.

File Name: Supplementary Movie 2
Description: The high-speed records of the PDA NPs removal with 8000 fps
